# Synthesis and Biological Evaluation of 3-Benzisoxazolyl-4-indolylmaleimides as Potent, Selective Inhibitors of Glycogen Synthase Kinase-3β

**DOI:** 10.3390/molecules18055498

**Published:** 2013-05-13

**Authors:** Qing Ye, Meng Li, Yubo Zhou, Tao Pang, Lei Xu, Jiayi Cao, Liang Han, Yujin Li, Weisi Wang, Jianrong Gao, Jia Li

**Affiliations:** 1 State Key Laboratory Breeding Base of Green Chemistry-Synthesis Technology, Zhejiang University of Technology, Hangzhou 310032, Zhejiang, China; E-Mails: yeqing1975@yeah.net (Q.Y.); limeng71085110@126.com (M.L.); hanliang@zjut.edu.cn (L.H.); lyjzjut@zjut.edu.cn (Y.L.); 2 The National Center for Drug Screening, Shanghai 201203, China; E-Mails: ybzhou@mail.shcnc.ac.cn (Y.Z.); tyne_pang@hotmail.com (T.P.); lxu@mail.shcnc.ac.cn (L.X.); jycao@mail.shcnc.ac.cn (J.C.); 3 ZJU-ENS Joint Laboratory of Medicinal Chemistry, College of Pharmaceutical Sciences, Zhejiang University, Hangzhou 310058, Zhejiang, China, E-Mail: pipilu1985@zju.edu.cn

**Keywords:** 3-benzisoxazolyl-4-indolylmaleimides, synthesis, GSK-3β, biological activity, docking, SAR

## Abstract

A series of novel 3-benzisoxazolyl-4-indolyl-maleimides were synthesized and evaluated for their GSK-3β inhibitory activity. Most compounds exhibited high inhibitory potency towards GSK-3β. Among them, compound **7j** with an IC_50_ value of 0.73 nM was the most promising GSK-3β inhibitor. Preliminary structure-activity relationships were examined and showed that different substituents on the indole ring and N^1^-position of the indole ring had varying degrees of influence on the GSK-3β inhibitory potency. Compounds **7c**, **7f**, **7j**–**l** and **7o**–**q** could obviously reduce Aβ-induced Tau hyperphosphorylation by inhibiting GSK-3β in a cell-based functional assay.

## 1. Introduction

Glycogen synthase kinase-3 (GSK-3) is a serine/threonine protein kinase which was identified in the late 1970s. Mammalian GSK-3 exists as two isoforms, GSK-3α and GSK-3β which share high homology at the catalytic domain but significantly differ in their N-terminal domain (84% overall and 98% in catalytic domain). Both isomers ubiquitously exist in cells and tissues and have similar biochemical properties [1,2,3,4]. GSK-3β plays a critical role in glycogen metabolism, embryogenesis, mitotic regulation, inflammation and neuroplasticity [5,6,7,8]. Inhibition of GSK-3β may provide therapy approach for several diseases such as cancer, type 2 diabetes, chronic inflammatory processes, stroke, bipolar disorders and Alzheimer's disease [9,10,11,12,13]. Accordingly, searching for novel and selective GSK-3β inhibitors is a very active area in both academic centers and pharmaceutical companies.

**Figure 1:**
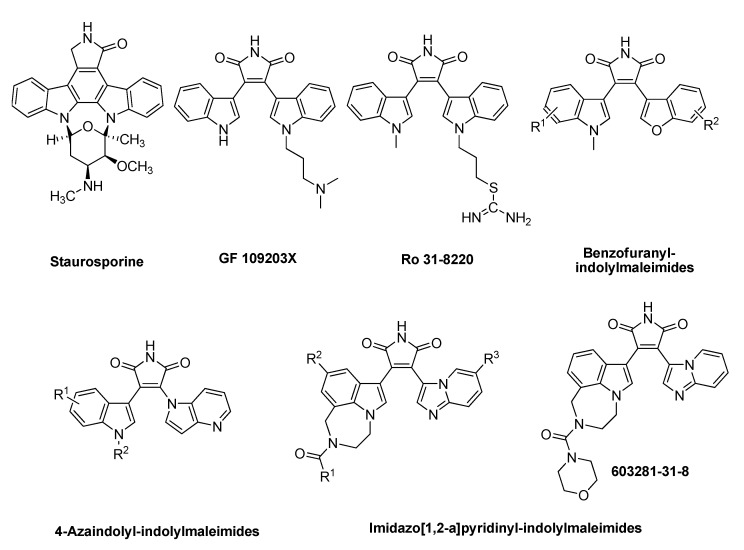
GSK-3β inhibitors.

Staurosporine, a microbial alkaloid, was identified as a potent but nonselective GSK-3β inhibitor. Various bisindolylmaleimides such as GF 109203X and Ro 31-8220 have also been developed as potent GSK-3β inhibitors based on staurosporine (Figure 1) [14,15,16]. However, most of these bisindolylmaleimides are not suitable for the treatment of diseases such as diabetes and Alzheimer’s disease due to their toxicity, poor solubility and low selectivity, especially against PKC family [17,18]. The replacement of one indole with other heteroaryl groups resulted in a series of monoindolylmaleimides such as 4-azaindolyl-indolyl-maleimides, benzofuranyl-indolyl-maleimides and imidazo[1,2-a]pyridinyl-indolyl-maleimides (Figure 1), which showed potent and selective GSK-3β inhibitory activities [19,20,21].

Among them, GSK-3β inhibitor **603281-31-8** developed by Eli Lilly & Co had reached preclinical studies for the treatment of diabetes and was proved efficacy in ZDF rats [19]. In view of these facts and also as a part of our work on the development of potent and selective GSK-3β inhibitors, herein we report the synthesis and biological evaluation of a new series of 3-benzisoxazolyl-4-indolyl-maleimides as GSK-3β inhibitors. Their structure-activity relationship and in silico molecular modeling study are also discussed in this study.

## 2. Results and Discussion

### 2.1. Chemistry

**Scheme 1:**
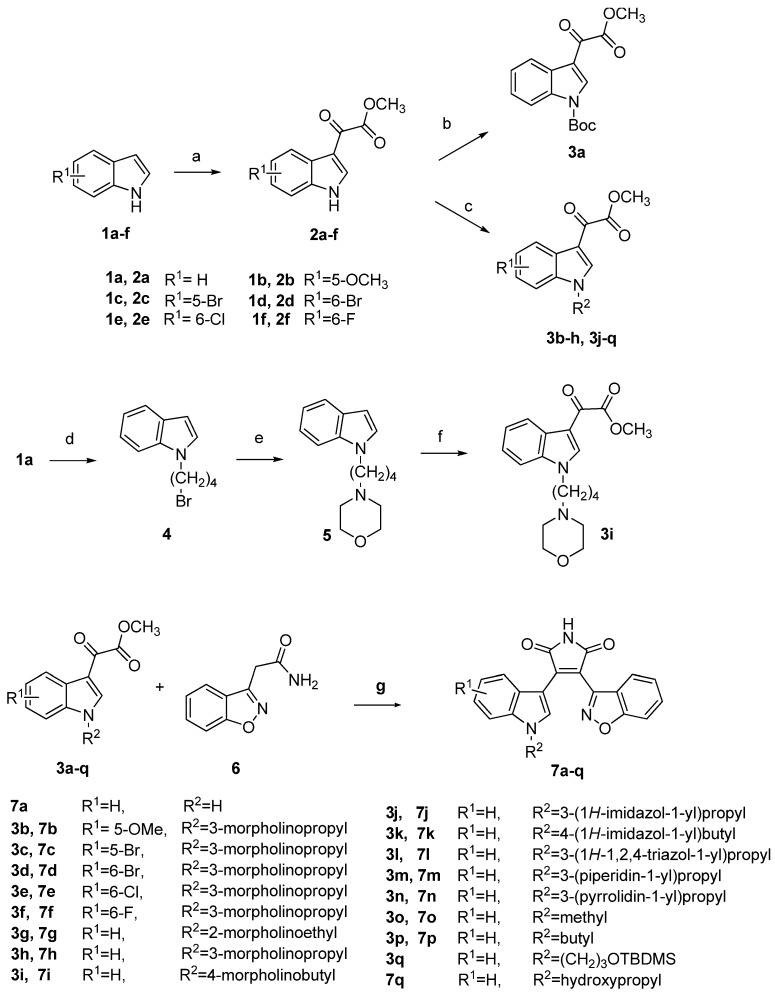
Synthetic route to compounds **7a**–**q**.

The general synthetic approach to target compounds **7a**–**q** is outlined in Scheme 1. 

*Reagents and conditions*: (**a**) i (COCl)_2_, Et_2_O; ii CH_3_ONa, CH_3_OH; (**b**) (Boc)_2_O, DMAP, THF; (**c**) NaH, DMF, R^2^X; (**d**) NaH, Br(CH_2_)_4_Br, DMF; (**e**) K_2_CO_3_, morpholine, DMF; (**f**) i HCl, (COCl)_2_, Et_2_O; ii CH_3_ONa, CH_3_OH; (**g**) i *t*-BuOK, THF; ii concentrated HCl.

Indole derivatives **1a**–**f **were reacted with oxalyl chloride in Et_2_O, followed by sodium methoxide to give compounds **2a**–**f**. Reaction of **2a **with (Boc)_2_O in the presence of a catalytic amount of DMAP in THF afforded **3a**. N-alkylation of **2a**–**f** with different alkyl halides resulted in key intermediates **3b**–**h** and ** 3j**–**q**. In addition, treatment of indole with 1,4-dibromobutane afforded **4**. N-substitution reaction of **4** with morpholine using K_2_CO_3_ as acid-trapping agent resulted in **5**, which was then treated with oxalyl chloride, followed by sodium methoxide to give another key intermediate **3i**. Condensation of glyoxylic esters **3a**–**q **with 2-(benzo[d]isoxazol-3-yl)acetamide **6** [22] in the presence of *t*-BuOK in THF afforded the target compounds **7a**–**q**.

### 2.2. Biological Activity and Molecular Modeling

#### 2.2.1. Enzymatic Activity

The GSK-3β inhibitory potency of all target compounds was examined. In addition, selected compounds **7c**, **7j** and **7o** were also tested for their inhibitory potency against other kinases (PKC- epsilon, JAK2, BRAF, IKK2, Drak2) to assess kinase selectivity. Staurosporine, a well known kinase inhibitor was used as the reference compound [19]. The results are listed in Table 1 and Table 2. As indicated in Table 1, most of the tested compounds showed similar or more potent GSK-3β inhibitory activity as compared to that of staurosporine. The potency of GSK-3β inhibition of tested compounds was mainly influenced by the substitutions on the indole ring and N^1^-position of the indole ring.

As expected, the introduction of suitable hydrophilic side chains at N^1^-position of the indole ring gave an obvious enhancement of GSK-3β inhibitory activity (*i.e*., **7a**, **7h**, **7j**–**l**, **7q**). Among them, compound **7j **with an imidazole group at the end of N^1^-propyl chain exhibited the most potent GSK-3β inhibitory activity, with an IC_50_ of 0.73 nM, which was about 460-fold more potent than that of compound **7a** (IC_50_ = 332.2 nM). Replacement of the terminal imidazole group in **7j** with other substituents such as 1,2,4-triazole (**7l**, IC_50_ = 20.9 nM), hydroxyl (**7q**, IC_50_ = 38.9 nM), piperidine (**7m**, IC_50_ = 511.8 nM) and pyrrolidine (**7o**, IC_50_ = 658.8 nM) resulted in a 29- to 915-fold less inhibitory potency. 

The results of inhibitory activities of compounds **7g**–**i**, **7j **and** 7k **showed that the length of the N^1^-alkyl linker affected GSK-3β inhibitory potency. For example, compound **7j** (IC_50_ = 0.73 nM) with a (CH_2_)_3_ linker showed better inhibitory activity than compound** 7k **(IC_50_ = 89.8 nM) with a (CH_2_)_4_ linker. The same conclusion could also be drawn from comparison of the inhibitory potency of **7h** and **7i**.

Interestingly, the introduction of a hydrophobic methyl group on the N^1^-position of the indole ring in **7a **resulted in a 15-fold increase in inhibitory potency for GSK-3β, while the replacement of the methyl with a large butyl group showed a 3-fold decrease in potency for GSK-3β inhibition.

When comparing the inhibitory activity of **7b**–**7f** with **7h**, it suggested that different substituents on the indole ring affected the inhibitory potency for GSK-3β. Compound **7c** (IC_50_ = 10.2 nM) with bromine at 5-position of the indole ring showed a 14-fold increase in inhibitory activity toward GSK-3β as compared to that of **7h** (IC_50_ = 137.7 nM). Fluorine at 6-position of the indole ring did not influence activity of **7h**, while bromine or chlorine at 6-position or methoxy at the 5-position showed less inhibitory potency.

**Table 1:** GSK-3β inhibitory activity of the target compounds. 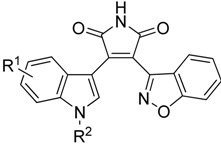

Compounds	R ^1^	R ^2^	IC_50_ (nM) ± SE ^a^
**Staurosporine**			72.2 ± 3.6
7a	H	H	332.2 ± 25.7
7b	5-OMe	3-morpholinopropyl	357.2 ± 17.1
7c	5-Br	3-morpholinopropyl	10.2 ± 5.9
7d	6-Br	3-morpholinopropyl	209.6 ± 8.9
7e	6-Cl	3-morpholinopropyl	510.0 ± 20.1
7f	6-F	3-morpholinopropyl	126.7 ± 3.2
7g	H	2-morpholinoethyl	615.6 ± 6.1
7h	H	3-morpholinopropyl	137.7 ± 6.0
7i	H	4-morpholinobutyl	410.7 ± 8.2
7j	H	3-(1*H*-imidazol-1-yl)propyl	0.73 ± 0.02
7k	H	4-(1*H*-imidazol-1-yl)butyl	89.8 ± 4.5
7l	H	3-(1*H*-1,2,4-triazol-1-yl)propyl	20.9 ± 5.1
7m	H	3-(piperidin-1-yl)propyl	511.8 ± 15.6
7n	H	3-(pyrrolidin-1-yl)propyl	658.8 ± 15.7
7o	H	methyl	22.1 ± 1.5
7p	H	butyl	58.7 ± 2.8
7q	H	hydroxypropyl	38.9 ± 2.6

^a^ SE: standard error mean.

**Table 2:** The selectivity to tested kinases of target compounds **7c**, **7j **and **7o**.

Kinases	IC_50_ (nM) ± SE ^a^ or inhibition% at 0.8 μg/mL (~2 μM)			
	Staurosporine	7c	7j	7o
**GSK-3β**	72.2 ±3.6	10.2 ± 5.9	0.7 ± 0.02	22.1 ± 1.5
**PKC-epsilon**	0.88 ± 0.03	12.1%	10.9%	21.2%
**JAK2**	2.26 ± 0.14	0.1%	4.9%	9.4%
**BRAF**	14.37 ± 0.86	3.4%	0.8%	1.1%
**IKK2**	1.21 ± 0.13	13.1%	3.5%	2.9%
**Drak2**	25.82 ± 2.11	1.67%	4.47%	13.76%

^a^ SE: standard error mean.

The data in Table 2 showed that staurosporine was a potent and nonselective kinase inhibitor as reported in the literature [14], which inhibits not only GSK-3β (IC_50_ = 72.2 nM) but also many other kinases (e.g., PKC-epsilon, JAK2, BRAF, IKK2 and Drak2). Selected compounds **7c**, **7j **and **7o** with high potency for GSK-3β inhibition were also evaluated for kinase selectivity against PKC-epsilon, JAK2, BRAF, IKK2 and Drak2. The results indicated that they displayed high selectivity for GSK-3β over other tested kinases.

#### 2.2.2. Cellular Activity

**Figure 2:**
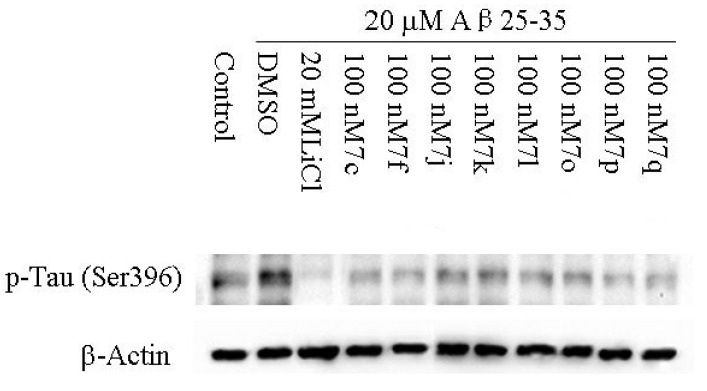
Effects of GSK-3β inhibitors on Tau phosphorylation (ser396) in SH-SY5Y cells.

It has been implicated that GSK-3β is involved in multiple cellular processes, and its ability to hyperphosphorylate Tau protein and induce neurofibrillary tangle was intensively studied. Therefore, the cell-based assay examining Tau phosphorylation at Serine 396 represents a direct functional assay to measure the cellular activity of GSK-3β inhibitors [23]. Compounds **7c**, **7f**, **7j**–**l **and **7o**–**q **were tested for the ability to reduce Tau phosphorylation at Ser 396 in human neuroblastoma SH-SY5Y cells. LiCl, a well-known inhibitor of GSK-3β [24], was used as a positive control in this assay. As shown in Figure 2, all selected compound significantly reduced Aβ_25-35_-induced Tau hyper-phosphorylation, showing that these compounds can inhibit GSK-3β activity at the cellular level. The Aβ-induced Tau hyperphosphorylation results in neurofibrillary tangle formation, which plays an important role in Alzheimer’s disease. Our data suggests that these novel GSK-3β inhibitors may have potential actions on inhibition of neurofibrillary tangle formation and would be tested for the treatment of Alzheimer’s disease. In addition, the predictions about these compounds’ brain permeability were performed using ADME module with Discovery Studio 2.1 software package. According to the prediction results (data not shown), most of them exhibited moderate blood-brain barrier permeability and would be further investigated for the treatment of Alzheimer’s disease.

#### 2.2.3. Molecular Modeling

**Figure 3:**
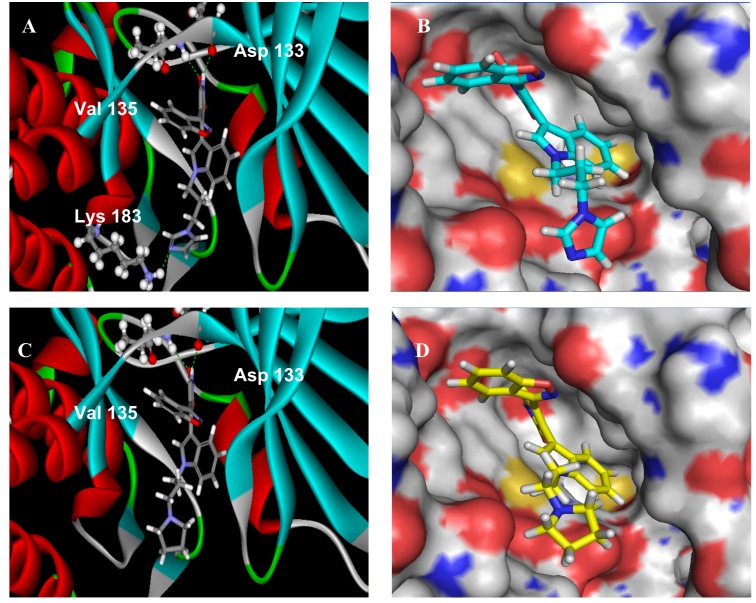
Docking of **7j** to GSK-3β crystal structure. (**A**) Ribbon show of compound **7j** bound to GSK-3β; (**B**) Surface show of compound **7j** dock into GSK-3β. Main text paragraph; (**C**) Ribbon show of compound **7n** bound to GSK-3β; (**D**) Surface show of compound **7n** dock into GSK-3β.

To examine possible binding modes of compounds bearing different side chains at N^1^-position of the indole ring (e.g., **7j **and **7n**) with GSK-3β, a docking analysis utilizing the C-DOCKER program within the Discovery Studio 2.1 software package was performed. The published X-ray crystal structure of GSK-3β (PDB ID: 1Q3D) [14] was used for the docking calculation. Figure 3 shows that both **7j **and **7n** could occupy the ATP binding site of GSK-3β with similar binding modes as a few other ATP-competitive inhibitors of GSK-3β [14,25], and they could thus serve as ATP-competitive inhibitors of GSK-3β. The NH and carbonyl group in maleimide ring of **7j** and **7n** could form two key hydrogen bonds with Asp133 and Val135 of GSK-3β. Besides, the 3-position nitrogen atom of the imidazole ring of **7j** could form another hydrogen bond with Lys-183, which was not observed in **7n**. Furthermore, the CDOCKERENERAGE of **7j** (−20.821 kcal/mol) was much lower than that of **7n** (2.971 kcal/mol). Thus, the molecular docking study results could explain the fact that **7j **showed significantly improved potency compared to that of **7n**.

## 3. Experimental

### 3.1. General

Melting points were determined with a BÜCHI Melting Point B-450 apparatus (Büchi Labortechnik, Flawil, Switzerland). The ^1^H-NMR spectra were recorded in DMSO-*d*_6_ or CDCl_3_ on Bruker Avance DMX 500 at 500 MHz (chemical shifts are expressed as *δ* values relative to TMS as internal standard). ESI spectra (positive ion mode) were recorded on an Esquire-LC-00075 spectrometer. Elemental analyses were performed on an Eager 300 instrument. All reactions were monitored by thin-layer chromatography (TLC). All reagents were obtained from commercial sources and used without further purification unless stated. Et_2_O and THF were distilled from sodium-benzophenone. DMF was distilled from calcium hydride.

### 3.2. General Procedure for the Preparation of ***2a**–**f***

Oxalyl chloride (3.40 g, 26 mmol) in Et_2_O (5 mL) was added dropwise to a solution of indole adducts **1a**–**f** (26 mmol) in Et_2_O (30 mL) at 0~5 °C [26,27]. The reaction mixture was stirred under the same conditions for 1 h, and a 20 wt% solution of CH_3_ONa in MeOH (14.05 g, 52 mmol) was added dropwise at −30 °C~−20 °C. After addition, the mixture was stirred for 30 min at room temperature, and poured into cold water (100 mL). The product was isolated with filtration, washed with dichloromethane and dried to afford **2a**–**f**.

*Methyl 2-(1H-indol-3-yl)-2-oxoacetate* (**2a**). Light yellow solid, 86.3% yield, mp: 208–210 °C. ^1^H-NMR (DMSO-*d*_6_): δ 12.48 (brs, 1H), 8.46 (d, *J* = 3.5 Hz, 1H), 8.16 (d, *J* = 7.0 Hz, 1H), 7.55 (d, *J* = 7.0 Hz, 1H), 7.32–7.26 (m, 2H), 3.90 (s, 3H). 

*Methyl 2-(5-methoxy-1H-indol-3-yl)-2-oxoacetate* (**2b**). Light yellow solid, 71.0% yield, mp: 221–223 °C. ^1^H-NMR (DMSO-*d*_6_): δ 12.34 (brs, 1H), 8.37 (d, *J* = 3.0 Hz, 1H), 7.66 (d, *J* = 2.0 Hz, 1H), 7.45 (d, *J* = 9.0 Hz, 1H), 6.93 (dd, *J* = 9.0, 2.0 Hz, 1H), 3.89 (s, 3H), 3.81 (s, 3H).

*Methyl 2-(5-bromo-1H-indol-3-yl)-2-oxoacetate* (**2c**). Light yellow solid, 55.3% yield, mp: 215–217 °C. ^1^H-NMR (DMSO-*d*_6_) δ 12.59 (brs, 1H), 8.52 (d, *J* = 3.0 Hz, 1H), 8.30 (d, *J* = 2.0 Hz, 1H), 7.54 (d, *J* = 8.5 Hz, 1H), 7.45 (dd, *J* =8.5, 2.0 Hz, 1H), 3.90 (s, 3H).

*Methyl 2-(6-bromo-1H-indol-3-yl)-2-oxoacetate* (**2d**). Light yellow solid, 58.9% yield, mp: 207–209 °C. ^1^H-NMR (DMSO-*d*_6_): δ 12.50 (brs, 1H), 8.50 (s, 1H), 8.10 (d, *J* = 8.5 Hz, 1H), 7.75 (d, *J* = 2.0 Hz, 1H), 7.43 (dd, *J* =8.5, 2.0 Hz, 1H), 3.90 (s, 3H).

*Methyl 2-(6-chloro-1H-indol-3-yl)-2-oxoacetate* (**2e**). Light yellow solid, 62.8% yield, mp: 246–248 °C. ^1^H-NMR (DMSO-*d*_6_): δ 12.52 (brs, 1H), 8.51 (d, *J* = 3.5 Hz, 1H), 8.15 (d, *J* = 8.5 Hz, 1H), 7.62 (d, *J* = 2.0 Hz, 1H), 7.31 (dd, *J* =8.5, 2.0 Hz, 1H), 3.90 (s, 3H).

*Methyl 2-(6-fluoro-1H-indol-3-yl)-2-oxoacetate* (**2f**). Light yellow solid, 60.3% yield, mp: 182–184 °C. ^1^H-NMR (DMSO-*d*_6_): δ 12.48 (brs, 1H), 8.48 (s, 1H), 8.15 (dd, *J* = 8.5, 5.5 Hz, 1H), 7.36 (dd, *J* = 9.5, 2.0 Hz, 1H), 7.15 (td, *J* = 9.5, 2.0 Hz, 1H), 3.90 (s, 3H).

### 3.3. tert-Butyl 3-(2-methoxy-2-oxoacetyl)-1H-indole-1-carboxylate (***3a***)

A solution of **2a **(2.0 g, 9.8 mmol), Boc anhydride (2.7 g, 12.3 mmol) and DMAP (0.01 g) in dry THF (100 mL) was reacted for 3 h at room temperature. The solvent was removed under vacuum, and the residue was recrystallized from petroleum ether/ethyl acetate to give 2.0 g (67.1%) **3a** as a white solid, mp: 132–133 °C. ^1^H-NMR (CDCl_3_) δ 8.81 (s, 1H), 8.40 (dd, *J* = 6.5, 2.0 Hz, 1H), 8.17 (dd, 1H, *J* = 6.5, 1.5 Hz, 1H), 7.41–7.38 (m, 2H), 3.98 (s, 3H), 1.71 (s, 9H). 

### 3.4. General Procedure for the Preparation of ***3b**–**h***, ***3j**–**n*** and ***3q***

70% NaH (0.51 g, 14.8 mmol) was added portionwise to a solution of **2a**–**f** (14.8 mmol) in DMF (30 mL) at 0~5 °C. The reaction mixture was warmed to room temperature and stirred for 30 min. After that, the appropriate R^2^X [4-(3-chloropropyl)morpholine, 4-(2-chloroethyl)morpholine, 1-(3-chloropropyl)-1*H*-imidazole, 1-(4-chlorobutyl)-1*H*-imidazole, 1-(3-chloropropyl)-1*H*-1,2,4-triazole, 1-(3-chloropropyl)piperidine, 1-(3-chloropropyl)pyrrolidine or (3-bromopropoxy)(*tert*-butyl)dimethyl-silane)] (19.2 mmol) was added. Then the mixture was headed to 55~60 °C and reacted for 12 h. After cooling, the mixture was poured into cold water (300 mL) and extracted with ethyl acetate (3 × 100 mL). The organic phase was combined, washed with brine (3 × 300 mL), dried over Na_2_SO_4_ and concentrated *in vacuo*. The residue was purified by flash column chromatography on silica gel using ethyl acetate/methanol (50:1, v/v) as eluent to afford **3b**–**h**, **3j**–**n **and **3q**.

*Methyl 2-(5-methoxy-1-(3-morpholinopropyl)-1H-indol-3-yl)-2-oxoacetate* (**3b**). Light yellow solid, 64.7% yield, mp: 67–68 °C. ^1^H-NMR (CDCl_3_): δ 8.38 (s, 1H), 7.95 (d, *J* = 2.5 Hz, 1H), 7.32 (d, *J* = 9.0 Hz, 1H), 6.97 (dd, *J* = 9.0, 2.5 Hz, 1H), 4.28 (t, *J* =7.0 Hz, 2H), 3.95 (s, 3H), 3.91 (s, 3H), 3.77–3.70 (m, 4H), 2.42–2.38 (m, 4H), 2.27 (t, *J* = 6.5 Hz, 2H), 2.06–2.01 (m, 2H). ESI-MS: *m/z* [M+H]^+^ 361. Anal. Calcd for C_19_H_24_N_2_O_5_: C, 63.32; H, 6.71; N, 7.77. Found: C, 63.49; H, 6.68; N, 7.49.

*Methyl 2-(5-bromo-1-(3-morpholinopropyl)-1H-indol-3-yl)-2-oxoacetate* (**3c**). Light yellow solid, 56.6% yield, mp: 112–113 °C. ^1^H-NMR (CDCl_3_) δ 8.43 (s, 1H), 8.31 (d, *J* = 8.5 Hz, 1H), 7.67 (d, *J* = 2.0 Hz, 1H), 7.45 (dd, *J* = 8.5, 2.0 Hz, 1H), 4.29 (t, *J* = 7.0 Hz, 2H), 3.95 (s, 3H), 3.81–3.75 (m, 4H), 2.43–2.39 (m, 4H), 2.25 (t, *J* = 6.5 Hz, 2H), 2.05–2.01 (m, 2H). ESI-MS: *m/z* [M+H]^+^ 409. Anal. Calcd for C_18_H_21_BrN_2_O_4_: C, 52.82; H, 5.17; N, 6.84. Found: C, 52.96; H, 5.23; N, 6.76.

*Methyl 2-(6-bromo-1-(3-morpholinopropyl)-1H-indol-3-yl)-2-oxoacetate* (**3d**). Light yellow solid, 54.9% yield, mp: 102–103 °C. ^1^H-NMR (CDCl_3_): δ 8.43 (s, 1H), 8.31 (d, *J* = 8.5 Hz, 1H), 7.67 (d, *J* = 1.5 Hz, 1H), 7.45 (dd, *J* = 8.5, 1.5 Hz, 1H), 4.29 (t, *J* = 6.5 Hz, 2H), 3.95 (s, 3H), 3.86–3.69 (m, 4H), 2.43–2.39 (m, 4H), 2.25 (t, *J* = 6.5 Hz, 2H), 2.05–2.01 (m, 2H). ESI-MS: *m/z* [M+H]^+^ 409. Anal. Calcd for C_18_H_21_BrN_2_O_4_: C, 52.82; H, 5.17; N, 6.84. Found: C, 52.68; H, 5.26; N, 6.68.

*Methyl 2-(6-chloro-1-(3-morpholinopropyl)-1H-indol-3-yl)-2-oxoacetate* (**3e**). Light yellow solid, 59.1% yield, mp: 115–116 °C. ^1^H-NMR (CDCl_3_): δ 8.42 (s, 1H), 8.34 (d, *J* = 8.5 Hz, 1H), 7.49 (d, *J* = 1.5 Hz, 1H), 7.30 (dd, *J* = 8.5, 1.5 Hz, 1H), 4.28 (t, *J* = 6.5 Hz, 2H), 3.94 (s, 3H), 3.80–3.70 (m, 4H), 2.43–2.37 (m, 4H), 2.24 (t, *J* = 6.5 Hz, 2H), 2.04–2.00 (m, 2H). ESI-MS: *m/z* [M+H]^+^ 365. Anal. Calcd for C_18_H_21_ClN_2_O_4_: C, 59.26; H, 5.80; N, 7.68. Found: C, 59.44; H, 5.78; N, 7.45.

*Methyl 2-(6-fluoro-1-(3-morpholinopropyl)-1H-indol-3-yl)-2-oxoacetate* (**3f**). Light yellow solid,64.8% yield, mp:116–117 °C. ^1^H-NMR (CDCl_3_): δ 8.44 (s, 1H), 8.38 (dd, *J* = 8.5, 5.5 Hz, 1H), 7.16 (dd, *J* = 9.0, 2.0 Hz, 1H), 7.13–7.06 (m, 1H), 4.27 (t, *J* = 6.5 Hz, 2H), 3.95 (s, 3H), 3.79–3.70 (m, 4H), 2.43–2.39 (m, 4H), 2.27 (t, *J* = 6.5 Hz, 2H), 2.06–2.00 (m, 2H). ESI-MS: *m/z* [M+H]^+^ 349. Anal. Calcd for C_18_H_21_FN_2_O_4_: C, 62.06; H, 6.08; N, 8.04. Found: C, 62.18; H, 6.23; N, 8.25.

*Methyl 2-(1-(2-morpholinoethyl)-1H-indol-3-yl)-2-oxoacetate* (**3g**). Light yellow solid, 52.4% yield, mp: 110–112 °C. ^1^H-NMR (CDCl_3_): δ 8.51 (s, 1H), 8.49–8.44 (m, 1H), 7.43–7.39 (m, 1H), 7.38–7.35 (m, 2H), 4.29 (t, *J* = 6.5 Hz, 2H), 3.97 (s, 3H), 3.75–3.69 (m, 4H), 2.81 (t, *J* = 6.5 Hz, 2H), 2.54–2.47 (m, 4H). ESI-MS: *m/z* [M+H]^+^ 317. Anal. Calcd for C_17_H_20_N_2_O_4_: C, 64.54; H, 6.37; N, 8.86. Found: C, 64.71; H, 6.68; N, 8.68.

*Methyl 2-(1-(3-morpholinopropyl)-1H-indol-3-yl)-2-oxoacetate* (**3h**). Light yellow solid, 67.5% yield, mp: 103–104 °C. ^1^H-NMR (CDCl_3_): δ 8.47–8.43 (m, 2H), 7.46–7.41 (m, 1H), 7.38–7.32 (m, 2H), 4.32 (t, *J* = 6.5 Hz, 2H), 3.95 (s, 3H), 3.78–3.72 (m, 4H), 2.44–2.38 (m, 4H), 2.28 (t, *J* = 6.5 Hz, 2H), 2.08–2.02 (m, 2H). ESI-MS: *m/z* [M+H]^+^ 331. Anal. Calcd for C_18_H_22_N_2_O_4_: C, 65.44; H, 6.71; N, 8.48. Found: C, 65.27; H, 6.68; N, 8.58.

*Methyl 2-(1-(3-(1H-imidazol-1-yl)propyl)-1H-indol-3-yl)-2-oxoacetate* (**3j**). Light yellow solid, 52.8% yield, mp: 72–73 °C. ^1^H-NMR (CDCl_3_) δ 8.51–8.46 (m, 1H), 8.36 (s, 1H), 7.53 (s, 1H), 7.42–7.34 (m, 2H), 7.30–7.27 (m, 1H), 7.17 (s, 1H), 6.96 (s, 1H), 4.20 (t, *J* = 7.0 Hz, 2H), 4.00 (t, *J* = 7.0 Hz, 2H), 3.98 (s, 3H), 2.48–2.40 (m, 2H). ESI-MS: *m/z* [M+H]^+^ 312. Anal. Calcd for C_17_H_17_N_3_O_3_: C, 65.58; H, 5.50; N, 13.50. Found: C, 65.59; H, 4.88; N, 13.68.

*Methyl 2-(1-(4-(1H-imidazol-1-yl)butyl)-1H-indol-3-yl)-2-oxoacetate* (**3k**). Light yellow solid, 41.6% yield, ^1^H-NMR (CDCl_3_) δ 8.50–8.42 (m, 1H), 8.35 (s, 1H), 7.44 (s, 1H), 7.38–7.32 (m, 3H), 7.06 (s, 1H), 6.85 (s, 1H), 4.19 (t, *J* = 7.0 Hz, 2H), 3.96 (s, 3H), 3.93 (t, *J* = 7.0 Hz, 2H), 1.95–1.86 (m, 2H), 1.87–1.77 (m, 2H). ESI-MS: *m/z* [M+H]^+^ 326. Anal. Calcd for C_18_H_19_N_3_O_3_: C, 66.45; H, 5.89; N, 12.91. Found: C, 66.62; H, 5.77; N, 12.85.

*Methyl 2-(1-(3-(1H-1,2,4-triazol-1-yl)propyl)-1H-indol-3-yl)-2-oxoacetate* (**3l**). Light yellow solid, 64.5% yield, mp: 98–99 °C. ^1^H-NMR (CDCl_3_) δ 8.49–8.45 (m, 1H), 8.44 (s, 1H), 8.08 (s, 1H), 8.05 (s, 1H), 7.40–7.36 (m, 2H), 7.35–7.32 (m, 1H), 4.31 (t, *J* = 7.0 Hz, 2H), 4.18 (t, *J* = 7.0 Hz, 2H), 3.97 (s, 3H), 2.59–2.49 (m, 2H). ESI-MS: *m/z* [M+H]^+^ 313. Anal. Calcd for C_16_H_16_N_4_O_3_: C, 61.53; H, 5.16; N, 17.94. Found: C, 61.69; H, 5.31; N, 17.99.

*Methyl 2-oxo-2-(1-(3-(piperidin-1-yl)propyl)-1H-indol-3-yl)acetate* (**3m**). Light yellow solid, 52.4% yield, mp: 66–67 °C. ^1^H-NMR (DMSO-*d*_6_) δ 8.47 (s, 1H), 8.20 (d, *J* = 7.5 Hz, 1H), 7.68 (d, *J* = 7.5 Hz, 1H), 7.39–7.27 (m, 2H), 4.34 (t, *J* = 7.0 Hz, 2H), 3.90 (s, 3H), 2.29–2.18 (m, 4H), 2.13 (t, *J* = 6.5 Hz, 2H), 1.96–1.90 (m, 2H), 1.55–1.41 (m, 4H), 1.40–1.30 (m, 2H). ESI-MS: *m/z* [M+H]^+^ 329. Anal. Calcd for C_19_H_24_N_2_O_3_: C, 69.49; H, 7.37; N, 8.53. Found: C, 69.69; H, 7.68; N, 8.48.

*Methyl 2-oxo-2-(1-(3-(pyrrolidin-1-yl)propyl)-1H-indol-3-yl)acetate* (**3n**). Light yellow solid, 48.5% yield, mp: 56–58 °C. ^1^H-NMR (DMSO-*d*_6_) δ 8.50 (s, 1H), 8.19 (d, *J* = 8.0 Hz, 1H), 7.69 (d, *J* = 8.0 Hz, 1H), 7.39–7.27 (m, 2H), 4.37 (t, *J* = 7.0 Hz, 2H), 3.90 (s, 3H), 2.42–2.39 (m, 4H), 2.35 (t, *J* = 6.5 Hz, 2H), 2.00–1.92 (m, 2H), 1.72–1.67 (m, 4H). ESI-MS: *m/z* [M+H]^+^ 311. Anal. Calcd for C_18_H_18_N_2_O_3_: C, 69.66; H, 5.85; N, 9.03. Found: C, 69.78; H,5.96; N, 9.22.

*Methyl 2-(1-(3-((tert-butyldimethylsilyl)oxy)propyl)-1H-indol-3-yl)-2-oxoacetate* (**3q**). Light yellow solid, 62.5% yield, mp: 69–71 °C. ^1^H-NMR (CDCl_3_): δ 8.40–8.36 (m, 1H), 8.31 (s, 1H), 7.39–7.34 (m, 1H), 7.31–7.24 (m, 2H), 4.27 (t, *J* = 7.0 Hz, 2H), 3.88 (s, 3H), 3.52 (t, *J* = 7.0 Hz, 2H), 2.06–1.92 (m, 2H), 0.87 (s, 9H), 0.01 (s, 6H). ESI-MS: *m/z* [M+H]^+^ 349. Anal. Calcd for C_20_H_29_NO_4_Si: C, 63.97; H, 7.78; N, 3.73. Found: C, 64.05; H, 7.86; N, 3.65.

### 3.5. 1-(4-Bromobutyl)-1H-indole (***4***)

70% NaH (1.77 g, 51.6 mmol) was added portionwise to a solution of indole (5.0 g, 43.0 mmol) in DMF (50 mL) at 0~5 °C. The mixture was warmed to room temperature and stirred for 30 min. After that, it was added dropwise to a mixture of 1,4-dibromobutane (46.4 g, 215 mmol) in DMF (10 mL) at room temperature, and stirred for 12 h. The mixture was then poured into cold water (300 mL) and extracted with ethyl acetate (3 × 100 mL). The organic phase was combined, washed with brine (3 × 300 mL), dried over Na_2_SO_4_ and concentrated *in vacuo*. The residue was purified by flash column chromatography on silica gel using petroleum ether/ethyl acetate (80:1, v/v) as eluent to afford 7.6 g (71.2%) of **4 **as a colorless liquid. ^1^H-NMR (CDCl_3_): δ 7.66 (d, *J* = 8.0 Hz, 1H), 7.36 (d, *J* = 8.0 Hz, 1H), 7.25–7.22 (m, 1H), 7.14–7.11 (m, 2H), 6.53 (d, *J* = 3.0 Hz, 1H), 4.19 (t, *J* = 7.0 Hz, 2H), 3.40 (t, *J* = 6.5 Hz, 2H), 2.07–2.00 (m, 2H), 1.90–1.85 (m, 2H).

### 3.6. 4-(4-(1H-Indol-1-yl)butyl)morpholine (***5***)

A mixture of **4 **(2.0 g, 7.9 mmol), morpholine (6.9 g, 79.0 mmol) and potassium carbonate (1.9 g, 13.8 mmol) in DMF (30 mL) was stirred at 50 °C for 6 h[28,29,30,31,32,33,34,35,36,37,38,39,40]. After cooling, the mixture was poured into cold water (200 mL) and extracted with ethyl acetate (3 × 100 mL). The organic phase was combined, washed with brine (3 × 300 mL), dried over Na_2_SO_4_ and concentrated in vacuum. The residue was purified by flash column chromatography on silica gel using petroleum ether/ethyl acetate/triethylamine (20:100:1, v/v/v) as eluent to afford 1.63 g (80.1%) of **5** as a colorless liquid, ^1^H-NMR (CDCl_3_): δ 7.65 (d, *J* = 8.0 Hz, 1H), 7.37 (d, *J* = 8.0 Hz, 1H), 7.24–7.21 (m, 1H), 7.14–7.11 (m, 2H), 6.51 (d, *J* = 3.0 Hz, 1H), 4.17 (t, *J* = 7.0 Hz, 2H), 3.75–3.70 (m, 4H), 2.39–2.33 (m, 6H), 1.93–1.87 (m, 2H), 1.57–1.50 (m, 2H). ESI-MS: *m/z* [M+H]^+^ 259. Anal. Calcd for C_16_H_22_N_2_O: C, 74.38; H, 8.58; N, 10.84. Found: C, 74.53; H, 8.64; N, 10.93.

### 3.7. Methyl 2-(1-(4-morpholinobutyl)-1H-indol-3-yl)-2-oxoacetate (***3i***)

1M HCl in dioxane (5.5 mL, 5.5 mmol) was added to a solution of **5** (1.29 g, 5.0 mmol) in CH_3_CN (30 mL) and Et_2_O (30 mL) at room temperature and stirred for 30 min. After that, oxalyl chloride (0.76 g, 6.0 mmol) in Et_2_O (5 mL) was added dropwise at 0~5 °C and the reaction mixture was stirred for 2 h at the same temperature. MeOH (10 mL) was added dropwise to the mixture at 0~5 °C and the result solution was stirred for 2 h at room temperature, then poured into a cold aqueous NaHCO_3_ solution and extracted with ethyl acetate (3 × 50 mL). The organic phase was combined, washed with brine (150 mL), dried over Na_2_SO_4_ and concentrated under vacuum. The residue was purified by flash column chromatography on silica gel using ethyl acetate/methanol (50:1, v/v) as eluent to afford 0.94 g (54.6%) of **3i** as a light yellow solid, mp: 84–85 °C. ^1^H-NMR (CDCl_3_): δ 8.48–8.44 (m, 1H), 8.40 (s, 1H), 7.44–7.38 (m, 1H), 7.36–7.34 (m, 2H), 4.23 (t, *J* = 7.5 Hz, 2H), 3.97 (s, 3H), 3.73–3.65 (m, 4H), 2.39–2.34 (m, 6H), 2.02–1.92 (m, 2H), 1.59–1.51(m, 2H). ESI-MS: *m/z* [M+H]^+^ 345. Anal. Calcd for C_19_H_24_N_2_O_4_: C, 66.26; H, 7.02; N, 8.13. Found: C, 66.40; H, 7.11; N, 8.32.

### 3.8. General Procedure for the Preparation of ***3o*** and ***3p***

70% NaH (0.17 g, 4.9 mmol) was added portionwise to a solution of **2a **(1.0 g, 4.9 mmol) in DMF (10 mL) at 0~5 °C. The reaction mixture was warmed to room temperature and stirred for 30 min. After that, iodomethane or 1-bromobutane (5.9 mmol) was added at 0 °C and stirred for 1 h at room temperature. The mixture was then poured into cold water (100 mL) and extracted with ethyl acetate (3 × 50 mL). The organic phase was combined, washed with brine (3 × 150 mL), dried over Na_2_SO_4_ and concentrated *in vacuo*. The residue was purified by flash column chromatography on silica gel using petroleum ether/ ethyl acetate (3:1, v/v) as eluent to afford **3o **and **3p**.

*Methyl 2-(1-methyl-1H-indol-3-yl)-2-oxoacetate* (**3o**). Light yellow solid, 76.8% yield, mp: 73–74 °C. ^1^H-NMR (CDCl_3_) δ 8.51–8.42 (m, 1H), 8.35 (s, 1H), 7.39–7.36 (m, 3H), 3.96 (s, 3H), 3.88 (s, 3H). ESI-MS: *m/z* [M+H]^+^ 218. Anal. Calcd for C_12_H_11_NO_3_: C, 66.35; H, 5.10; N, 6.45. Found: C, 66.55; H, 5.20; N, 6.59.

*Methyl 2-(1-butyl-1H-indol-3-yl)-2-oxoacetate* (**3p**). White solid, 80.5% yield, mp: 81–82 °C. ^1^H-NMR (CDCl_3_) δ 8.48–8.44 (m, 1H), 8.39 (s, 1H), 7.45–7.38 (m, 1H), 7.37–7.32 (m, 2H), 4.20 (t, *J* = 7.5 Hz, 2H), 3.96 (s, 3H), 1.86–1.82 (m, 2H), 1.42–1.38 (m, 2H), 0.98 (t, *J* = 7.5 Hz, 3H). ESI-MS: *m/z* [M+H]^+^ 260. Anal. Calcd for C_15_H_17_NO_3_: C, 69.48; H, 6.61; N, 5.40. Found: C, 69.62; H,6.52; N, 5.65.

### 3.9. General Procedure for the Preparation of ***7a**–**q***

A solution of t-BuOK (94.0 mg, 0.84 mmol) in THF (5mL) was added dropwise to a solution of **3a**–**q** (0.36 mmol) and 2-(benzo[d]isoxazol-3-yl)acetamide **6** (0.28 mmol) in dry THF (10 mL) at −10~0 °C. After stirring for 2 h at room temperature, concentrated hydrochloric acid (5 mL) was added and the result mixture was stirred for 30 min at room temperature, then poured into 10% NaHCO_3_ aqueous solution (100 mL) and extracted with ethyl acetate (3 × 50 mL). The organic phase was combined and washed with brine (3 × 150 mL), dried over Na_2_SO_4_ and concentrated *in vacuo*. The residue was purified by flash column chromatography on silica gel using dichloromethane/methanol (50:1, v/v) as eluent to afford **7a**–**q**. 

*3-(Benzo[d]isoxazol-3-yl)-4-(1H-indol-3-yl)-1H-pyrrole-2,5-dione* (**7a**). Red solid, 44.4% yield, mp: 227–228 °C. ^1^H-NMR (DMSO-*d*_6_) δ 12.14 (brs,1H), 11.44 (brs, 1H), 8.23 (s, 1H), 7.83 (d, *J* = 8.5 Hz, 1H), 7.69–7.58 (m, 2H), 7.44 (d, *J* = 8.0 Hz, 1H), 7.31 (t, *J* = 7.5 Hz, 1H), 7.08–7.04 (m, 1H), 6.72 (t, *J* = 8.0 Hz, 1H), 6.51 (d, *J* = 8.5 Hz, 1H). ESI-MS: *m/z* [M+H]^+^ 330. Anal. Calcd for C_19_H_11_N_3_O_3_: C, 69.30; H, 3.37; N, 12.76. Found: C, 69.06; H, 3.45; N, 12.64

*3-(Benzo[d]isoxazol-3-yl)-4-(5-methoxy-1-(3-morpholinopropyl)-1H-indol-3-yl)-1H-pyrrole-2,5-dione* (**7b**). Saffron yellow solid, 11.6% yield, mp: 191–193 °C. ^1^H-NMR (CDCl_3_) δ 8.27 (s, 1H), 7.97 (brs, 1H), 7.66–7.62 (m, 2H), 7.57 (td, *J* = 8.0, 1.5 Hz, 1H), 7.25–7.23 (m, 2H), 6.76 (dd, *J* = 9.0, 2.5 Hz, 1H), 5.94 (d, *J* = 2.5 Hz, 1H), 4.29 (t, *J* = 6.5 Hz, 2H), 3.77 (t, *J* = 4.5 Hz, 4H), 3.11 (s, 3H), 2.48–2.42 (m, 4H), 2.32 (t, *J* = 6.5 Hz, 2H), 2.06–2.01(m, 2H). ESI-MS: *m/z* [M+H]^+^ 487. Anal. Calcd for C_27_H_26_N_4_O_5_: C, 66.65; H, 5.39; N, 11.52. Found: C, 66.76; H, 5.47; N, 11.62. 

*3-(Benzo[d]isoxazol-3-yl)-4-(5-bromo-1-(3-morpholinopropyl)-1H-indol-3-yl)-1H-pyrrole-2,5-dione* (**7c**). Saffron yellow solid, 21.3% yield, mp: 180–182 °C. ^1^H-NMR (CDCl_3_) δ 8.32 (s, 1H), 7.97 (brs, 1H), 7.68 (t, *J* = 8.5 Hz, 2H), 7.61 (td, *J* = 8.5, 1.5 Hz, 1H), 7.32 (t, *J* = 8.0 Hz, 1H), 7.26–7.25 (m, 2H), 6.81 (s, 1H), 4.29 (t, *J* = 7.0 Hz, 2H), 3.75 (t, *J* = 4.5 Hz , 4H), 2.42–2.39 (m, 4H), 2.30 (t, *J* = 6.6 Hz, 2H), 2.03–2.01 (m, 2H). ESI-MS: *m/z* [M+H]^+^ 535. Anal. Calcd for C_26_H_23_N_4_O_4_Br: C, 58.33; H, 4.33; N, 10.46. Found: C, 58.21; H, 4.49; N, 10.53.

*3-(Benzo[d]isoxazol-3-yl)-4-(6-bromo-1-(3-morpholinopropyl)-1H-indol-3-yl)-1H-pyrrole-2,5-dione* (**7d**). Saffron yellow solid, 12.7% yield, mp: 226–228 °C. ^1^H-NMR (CDCl_3_) δ 8.27 (s, 1H), 7.97 (brs, 1H), 7.68–7.64 (m, 2H), 7.62 (d, *J* = 2.0 Hz, 1H), 7.59 (td, *J* = 7.0, 1.0 Hz, 2H), 7.29 (t, *J* = 8.0 Hz, 1H), 6.99 (dd, *J* = 8.0, 2.0Hz, 1H), 6.59 (d, *J* = 8.0 Hz, 1H), 4.29 (t, *J* = 6.5 Hz, 2H), 3.79 (t, *J* = 4.5 Hz, 4H), 2.47–2.40 (m, 4H), 2.29 (t, *J* = 6.5Hz, 2H), 2.05–2.02 (m, 2H). ESI-MS: *m/z* [M+H]^+^ 535. Anal. Calcd for C_26_H_23_N_4_O_4_Br: C, 58.33; H, 4.33; N, 10.46. Found: C, 58.39; H, 4.51; N, 10.42.

*3-(Benzo[d]isoxazol-3-yl)-4-(6-chloro-1-(3-morpholinopropyl)-1H-indol-3-yl)-1H-pyrrole-2,5-dione* (**7e**). Red solid, 11.5% yield, mp: 216–218 °C. ^1^H-NMR (CDCl_3_) δ 8.29 (s, 1H), 7.97 (brs, 1H), 7.65 (t, *J* = 7.5 Hz, 2H), 7.59 (t, *J* = 8.0 Hz, 1H), 7.45 (d, *J* = 1.5 Hz, 1H), 7.28 (t, *J* = 7.5 Hz, 1H), 6.85 (dd, *J* = 8.5, 1.5 Hz, 1H), 6.64 (d, *J* = 8.5 Hz, 1H), 4.29 (t, *J* = 6.5 Hz, 2H), 3.78 (t, *J* = 4.5 Hz, 4H), 2.45–2.41 (m, 4H), 2.29 (t, *J* = 6.5 Hz, 2H), 2.06–2.02 (m, 2H). ESI-MS: *m/z* [M+H]^+^ 491. Anal. Calcd for C_26_H_23_N_4_O_4_Cl: C, 63.61; H, 4.72; N, 11.41. Found: C, 63.52; H, 4.64; N, 11.51.

*3-(Benzo[d]isoxazol-3-yl)-4-(6-fluoro-1-(3-morpholinopropyl)-1H-indol-3-yl)-1H-pyrrole-2,5-dione* (**7f**). Yellow solid, 29.9% yield, mp: 219–221 °C. ^1^H-NMR (CDCl_3_) δ 8.29 (s, 1H), 8.06 (brs, 1H), 7.66–7.66 (m, 2H), 7.58 (t, *J* = 7.5 Hz, 1H), 7.29 (t, *J* = 7.5 Hz, 1H), 7.11 (d, *J* = 9.5 Hz, 1H), 6.68–6.62 (m, 2H), 4.27 (t, *J* = 6.5 Hz, 2H), 3.77 (t, *J* = 4.5 Hz, 4H), 2.46–2.41 (m, 4H), 2.31 (t, *J* = 6.5 Hz, 2H), 2.06–2.03 (m, 2H). ESI-MS: *m/z* [M+H]^+^ 475. Anal. Calcd for C_26_H_23_N_4_O_4_F: C, 65.81; H, 4.89; N, 11.81. Found: C, 65.99; H, 4.75; N, 11.87.

*3-(Benzo[d]isoxazol-3-yl)-4-(1-(2-morpholinoethyl)-1H-indol-3-yl)-1H-pyrrole-2,5-dione* (**7g**). Yellow solid, 24.0% yield, mp: 205–207 °C. ^1^H-NMR (CDCl_3_) δ 8.39 (s, 1H), 7.79 (brs, 1H), 7.57 (t, *J* = 7.5 Hz, 2H), 7.35 (d, *J* = 8.0 Hz, 1H), 7.25 (t, *J* = 7.5 Hz, 1H), 7.19 (t, *J* = 7.5 Hz, 1H), 6.90 (t, *J* = 7.5 Hz, 1H), 6.73 (d, *J* = 8.0 Hz, 1H), 4.31 (t, *J* = 6.5Hz, 2H), 3.73 (t, *J* = 7.5 Hz , 4H), 2.82 (t, *J* = 6.5 Hz, 2H), 2.56–2.42 (m, 4H). ESI-MS: *m/z* [M+H]^+^ 443. Anal. C_25_H_22_N_4_O_4_: C, 67.86; H, 5.01; N, 12.66. Found: C, 67.96; H, 5.11; N, 12.85. 

*3-(Benzo[d]isoxazol-3-yl)-4-(1-(3-morpholinopropyl)-1H-indol-3-yl)-1H-pyrrole-2,5-dione* (**7h**). Red solid, 38.1% yield, mp: 197–198 °C. ^1^H-NMR (CDCl_3_) δ 8.89 (brs, 1H), 8.33 (s, 1H), 7.66–7.60 (m, 2H), 7.55 (td, *J* = 7.0, 1.0 Hz 1H), 7.37 (d, *J* = 8.0 Hz, 1H), 7.24 (t, *J* = 7.5 Hz, 1H), 7.16 (t, *J* = 7.5 Hz, 1H), 6.86 (t, *J* = 7.5 Hz, 1H), 6.67 (d, *J* = 8.0 Hz, 1H), 4.32 (t, *J* = 6.5 Hz, 2H), 3.79 (t, *J* = 4.5 Hz, 4H), 2.52–2.42 (m, 4H), 2.37 (t, *J* = 7.0 Hz, 2H), 2.13–2.05 (m, 2H). ESI-MS: *m/z* [M+H]^+^ 457. Anal. C_26_H_24_N_4_O_4_: C, 68.41; H, 5.30; N, 12.27. Found: C, 68.69; H, 5.25; N, 12.41.

*3-(Benzo[d]isoxazol-3-yl)-4-(1-(4-morpholinobutyl)-1H-indol-3-yl)-1H-pyrrole-2,5-dione* (**7i**). Red solid, 22.6% yield, mp: 89–91 °C. ^1^H-NMR (CDCl_3_) δ 8.28 (s, 1H), 8.25(brs, 1H), 7.66–7.62 (m, 2H), 7.56 (t, *J* = 7.5 Hz, 1H), 7.35 (d, *J* = 8.0 Hz, 1H), 7.24 (t, *J* = 8.0 Hz, 1H), 7.18 (t, *J* = 7.0 Hz, 1H), 6.88 (t, *J* = 7.5 Hz, 1H), 6.72 (d, *J* = 8.0 Hz, 1H), 4.23 (t, *J* = 6.5 Hz, 2H), 3.71 (t, *J* = 4.5 Hz, 4H), 2.45–2.35 (m, 6H), 2.01–1.90 (m, 2H), 1.62–1.55 (m, 2H). ESI-MS: *m/z* [M+H]^+^ 471. Anal. C_27_H_26_N_4_O_4_: C, 68.92; H, 5.57; N, 11.91. Found: C, 68.75; H, 5.62; N, 11.82.

*3-(1-(3-(1H-Imidazol-1-yl)propyl)-1H-indol-3-yl)-4-(benzo[d]isoxazol-3-yl)-1H-pyrrole-2,5-dione* (**7j**). Red solid, 16.1% yield, mp: 208–210 °C. ^1^H-NMR (CDCl_3_) δ 8.32 (brs, 1H), 8.24 (s, 1H), 7.67 (d, *J* = 8.5 Hz,1H), 7.85 (d, *J* = 8.5 Hz,1H), 7.61–7.53 (m, 2H), 7.29 (t, *J* = 7.0 Hz, 1H), 7.24–7.18 (m, 2H), 7.17 (s, 1H), 6.98 (s, 1H), 6.94 (td, *J* = 7.0, 1.0 Hz, 1H), 6.81 (d, *J* = 8.0 Hz, 1H), 4.20 (t, *J* = 6.5 Hz, 2H), 3.99 (t, *J* = 6.5 Hz, 2H), 2.47–2.40 (m, 2H). ESI-MS: *m/z* [M+H]^+^ 438. Anal. C_25_H_19_N_5_O_3_: C, 68.64; H, 4.38; N, 16.01. Found: C, 68.81; H, 4.35; N, 16.26.

*3-(1-(4-(1H-Imidazol-1-yl)butyl)-1H-indol-3-yl)-4-(benzo[d]isoxazol-3-yl)-1H- pyrrole-2,5-dione* (**7k**). Red solid, 15.6% yield, mp: 203–205 °C. ^1^H-NMR (CDCl_3_) δ 8.35 (brs, 1H), 8.24 (s, 1H), 7.69–7.63 (m, 2H), 7.57 (td, *J* = 7.0, 1.5Hz, 1H), 7.47 (s, 1H), 7.29 (d, *J* = 6.0 Hz, 1H), 7.25 (d, *J* = 7.5 Hz, 1H), 7.19 (t, *J* = 7.5 Hz, 1H), 7.08 (s, 1H), 6.92 (t, *J* = 7.5 Hz, 1H), 6.87 (s, 1H), 6.78(d, *J* = 8.0 Hz, 1H), 4.21 (t, *J* = 6.5 Hz, 2H), 3.92 (t, *J* = 6.5 Hz, 2H), 1.95–1.87 (m, 2H), 1.86–1.80 (m, 2H). ESI-MS: *m/z* [M+H]^+^ 452. Anal. C_26_H_21_N_5_O_3_: C, 69.17; H, 4.69; N, 15.51. Found: C, 68.96; H, 4.65; N, 15.73.

*3-(1-(3-(1H-1,2,4-Triazol-1-yl)propyl)-1H-indol-3-yl)-4-(benzo[d]isoxazol-3-yl)-1H-pyrrole-2,5-dione* (**7l**). Red solid, 21.3% yield, mp: 214–216 °C. ^1^H-NMR (DMSO-*d*_6_) δ 11.46 (brs, 1H), 8.54 (s, 1H), 8.28 (s, 1H), 8.01 (s, 1H), 7.81 (d, *J* = 8.5 Hz, 1H), 7.66–7.61(m, 2H), 7.52 (d, *J* = 8.0 Hz, 1H), 7.29 (t, *J* = 7.5 Hz, 1H), 7.13 (t, *J* = 7.5 Hz, 1H), 6.79 (t, *J* = 8.0 Hz, 1H), 6.57 (d, *J* = 8.0 Hz, 1H), 4.36 (t, *J* = 6.5 Hz, 2H), 4.20 (t, *J* = 6.5 Hz, 2H), 2.36–2.28 (m, 2H). ESI-MS: *m/z* [M+H]^+^ 439. Anal. C_24_H_18_N_6_O_3_: C, 65.75; H, 4.14; N, 19.17. Found: C, 65.58; H, 4.12; N, 19.34.

*3-(Benzo[d]isoxazol-3-yl)-4-(1-(3-(piperidin-1-yl)propyl)-1H-indol-3-yl)-1H-pyrrole-2,5-dione* (**7m**). Red solid, 15.6% yield, mp: 73–75 °C. ^1^H-NMR (CDCl_3_) δ 8.32 (s, 1H), 7.64 (d, *J* = 8.5 Hz, 1H), 7.59 (d, *J* = 8.0 Hz, 1H), 7.55 (td, *J* = 8.0, 1.0 Hz, 1H), 7.38 (d, *J* = 8.5 Hz, 1H), 7.23 (t, *J* = 8.0 Hz, 1H), 7.16 (t, *J* = 8.0 Hz, 1H), 6.85 (t, *J* = 8.0 Hz, 1H), 6.67 (d, *J* = 8.0 Hz, 1H), 4.31 (t, *J* = 7.0 Hz, 2H), 2.55–2.35 (m, 6H), 2.17–2.10 (m, 2H), 1.73–1.61 (m, 4H), 1.52–1.47 (m, 2H). ESI-MS: *m/z* [M+H]^+^ 455. Anal. C_27_H_26_N_4_O_3_: C, 71.35; H, 5.77; N, 12.33. Found: C, 71.13; H, 5.63; N, 12.46.

*3-(Benzo[d]isoxazol-3-yl)-4-(1-(3-(pyrrolidin-1-yl)propyl)-1H-indol-3-yl)-1H-pyrrole-2,5-dione* (**7n**). Saffron yellow solid, 20.8% yield, mp: 171–173 °C. ^1^H-NMR (CDCl_3_) δ 8.41 (s, 1H), 7.63 (d, *J* = 8.5, 1H), 7.59 (d, *J* = 8.5, 1H), 7.54 (td, *J* = 8.5, 1H, 1H), 7.36 (d, *J* = 8.0 Hz, 1H), 7.22 (t, *J* = 7.5 Hz, 1H), 7.15 (t, *J* = 7.5 Hz, 1H), 6.83 (t, *J* = 7.5 Hz, 1H), 6.62 (d, *J* = 8.5 Hz, 1H), 4.32 (t, *J* = 7.0 Hz, 2H), 2.65–2.55 (m, 6H), 2.25–2.15 (m, 2H), 1.89–1.83 (m, 4H). ESI-MS: *m/z* [M+H]^+^ 441. Anal. C_26_H_24_N_4_O_3_: C, 70.89; H, 5.49; N, 12.72. Found: C, 70.68; H, 5.34; N, 12.97.

*3-(Benzo[d]isoxazol-3-yl)-4-(1-methyl-1H-indol-3-yl)-1H-pyrrole-2,5-dione* (**7o**). Red solid, 27.8% yield, mp: 221–222 °C. ^1^H-NMR (CDCl_3_) δ 8.24 (s, 1H), 7.81 (brs, 1H), 7.65 (d, *J* = 9.5 Hz, 2H), 7.57 (t, *J* = 8.0 Hz, 1H), 7.32 (d, *J* = 8.5 Hz, 1H), 7.19 (t, *J* = 8.0 Hz, 1H), 6.97 (t, *J* = 8.0 Hz, 1H), 6.88 (t, *J* = 7.5 Hz, 1H), 6.65 (d, *J* = 8.5 Hz, 1H), 3.90 (s, 3H). ESI-MS: *m/z* [M+H]^+^ 344. Anal. C_20_H_13_N_3_O_3_: C, 69.96; H, 3.82; N, 12.24. Found: C, 70.11; H, 3.77; N, 12.05.

*3-(Benzo[d]isoxazol-3-yl)-4-(1-butyl-1H-indol-3-yl)-1H-pyrrole-2,5-dione*(**7p**). Saffron yellow solid, 35.2% yield, mp: 180–182 °C. ^1^H-NMR (CDCl_3_) δ 8.25 (s, 1H), 7.82 (brs,1H), 7.65 (d, *J* = 8.5, 1H), 7.61 (d, *J* =8.5, 1H), 7.56 (t, *J* = 8.0 Hz, 1H), 7.34 (d, *J* = 8.0 Hz, 1H), 7.24 (t, *J* = 7.5 Hz, 1H), 7.18 (t, *J* = 7.5 Hz, 1H), 6.89 (t, *J* = 7.5 Hz, 1H), 6.75 (d, *J* =7.5 Hz, 1H), 4.22 (t, *J* = 7.5, 2H), 1.95–1.83 (m, 2H), 1.42–1.37 (m, 2H), 0.98 (t, *J* = 7.5 Hz, 3H). ESI-MS: *m/z* [M+H]^+^ 386. Anal. C_23_H_19_N_3_O_3_: C, 71.67; H, 4.97; N, 10.90. Found: C, 71.55; H, 4.84; N, 11.03.

*3-(Benzo[d]isoxazol-3-yl)-4-(1-(3-hydroxypropyl)-1H-indol-3-yl)-1H-pyrrole-2,5-dione* (**7q**). Yellow solid, 10.0% yield, mp: 219–220 °C. ^1^H-NMR (CDCl_3_) δ 8.29 (s, 1H), 7.64 (t, *J* = 8.0 Hz, 2H), 7.57 (t, *J* = 7.5 Hz, 1H), 7.40 (d, *J* = 8.5 Hz, 1H), 7.24 (t, *J* = 8.0 Hz, 1H), 7.19 (t, *J* = 8.0 Hz, 1H), 6.91 (t, *J* = 7.5 Hz, 1H), 6.79 (d, *J* = 7.5 Hz, 1H), 4.39 (t, *J* =7.0 Hz, 2H), 3.67 (t, *J* = 7.0 Hz, 2H), 2.17–2.10 (m, 2H). ESI-MS: *m/z* [M+H]^+^ 388. Anal. C_22_H_17_N_3_O_4_: C, 68.21; H, 4.42; N, 10.85. Found: C, 68.07; H, 4.51; N, 11.09.

### 3.10. Biological Activity Assay

#### 3.10.1. Kinase Assay

Human kinome, consisting of 518 kinases, is classified into CMGC, AGC, TK, TKL, CAMK, STE and other 7 subfamilies, according to the DNA sequence and evolution. GSK3β belongs to CMGC family. Here, the other family members were used to evaluate the selectivity of GSK3β inhibitors, including PKC-epsilon (AGC family), JAK2 (TK family), Braf (TKL family), DRAK2 (CAMK family) and IKKβ (other family).The recombinant GST-GSK-3βprotein was expressed in *Escherichia coli* strain BL21-Codon Plus (DE3), purified by GSTrap affinity chromatography, and cleaved by thrombin. The GSK-3β kinase assay was carried out with the Z´-LYTETM Kinase Assay kit Ser/Thr 9 Peptide substrate (Invitrogen, Grand, NY, USA) in 10 μL reaction volume containing 50 nM enzyme, 30 μM ATP and 2 μM substrate peptide. Drak2 Proteins were presented by professor Jiang-ping Wu (Laboratory of Immunology, Research Centre, CHUM, Notre Dame Hospital, Pavilion DeSève). The Drak2 kinase reaction was performed in a final assay volume of 3.4 μL using the ADP-GLO Kinase Assay Kit (Promega, Madison, WI, USA), according the ADP-GLO protocol and read on an EnVision plate reader. The recombinant PKC-epsilon, IKKβ, and JAK2 with N-terminal His-tag were expressed using baculovirus expression system and purified with Ni-Beads. BRAF protein was from Carna Biosciences, Inc. (Kobe Port Island, Japan). And the related kinase reactions were performed in a final assay volume of 10 μL using the related HTRF Assay Kit (Cisbio, Codolet, France). Reactions were according the HTRF protocol and read on an EnVision plate reader. All reactions were carried out in triplicate. IC_50_ values (concentration at which a 50% of enzyme inhibition is shown) were derived from a nonlinear regression model (curvefit) based on sigmoidal dose response curve (variable slope) and computed using Graphpad Prism version 5.02, (Graphpad Software, La Jolla, CA, USA). Data were expressed as mean ± SE. 

#### 3.10.2. Cell Culture and Western Blotting

SH-SY5Y human neuroblastoma cells were obtained from the American Type Culture Collection (ATCC, Manassas, VA, USA). Cells were cultured in 1:1 DMEM:Ham's F12 containing 10% (v/v) fetal bovine serum (HyClone, Logan, UT, USA), 1% penicillin, and 1% streptomycin at a humidified atmosphere with 5% CO_2_. The medium was changed every 2 days. For experiments, cells were and grown in 12-well plates until ~80% confluence, serum-deprived for 12 hours, incubated with GSK-3β inhibitors for 1 hour and Aβ_25–35_ (amyloid beta peptide 25–35, Sigma, St. Louis, MO, USA) for another 6 hours. Cells were rinsed twice with ice-cold PBS and lysed with 1 × SDS loading buffer. Samples were electrophoresed on 10% SDS-polyacrylamide gels, and transferred onto PVDF membranes. The membranes were blocked for 1 h with 5% (w/v) milk, incubated with rabbit anti-Tau [pS396] phosopho-specific antibody (Abcam, Cambridge, UK) for 2 h and the anti-rabbit secondary antibody for 1 h. Antigen-antibody complexes were detected by the ECL Kit.

## 4. Conclusions

In conclusion, a series of novel 3-benzisoxazolyl-4-indolyl-maleimides were synthesized and identified as potent and selective GSK-3β inhibitors. Among them, compound **7j** was the most promising GSK-3β inhibitor, with an IC_50_ of 0.73 nM, which was about 140-fold more potent than staurosporine. Futher cell-based functional assays showed that all selected compounds such as **7c**, **7f**, **7j**–**l** and **7o**–**q **could obviously reduce Tau phosphorylation at 100 nM by inhibiting GSK-3β. Preliminary SAR and molecular modeling studies provided further insights into interactions between GSK-3β and its inhibitors. The results are useful for the design of novel and selective GSK-3β inhibitors. Future progress on related series will be reported in due course. 
